# How plants handle multiple stresses: hormonal interactions underlying responses to abiotic stress and insect herbivory

**DOI:** 10.1007/s11103-016-0481-8

**Published:** 2016-04-19

**Authors:** Duy Nguyen, Ivo Rieu, Celestina Mariani, Nicole M. van Dam

**Affiliations:** Molecular Plant Physiology, Institute for Water and Wetland Research (IWWR), Radboud University, PO Box 9010, 6500 GL Nijmegen, The Netherlands; German Centre for Integrative Biodiversity Research (iDiv) Halle-Jena-Leipzig, Deutscher Platz 5e, 04103 Leipzig, Germany; Institute of Ecology, Friedrich Schiller University Jena, Dornburger-Str. 159, 07743 Jena, Germany

**Keywords:** Drought, Flooding, Herbivory, Hormonal cross-talk, Induced resistance, Stress responses

## Abstract

Adaptive plant responses to specific abiotic stresses or biotic agents are fine-tuned by a network of hormonal signaling cascades, including abscisic acid (ABA), ethylene, jasmonic acid (JA) and salicylic acid. Moreover, hormonal cross-talk modulates plant responses to abiotic stresses and defenses against insect herbivores when they occur simultaneously. How such interactions affect plant responses under multiple stresses, however, is less understood, even though this may frequently occur in natural environments. Here, we review our current knowledge on how hormonal signaling regulates abiotic stress responses and defenses against insects, and discuss the few recent studies that attempted to dissect hormonal interactions occurring under simultaneous abiotic stress and herbivory. Based on this we hypothesize that drought stress enhances insect resistance due to synergistic interactions between JA and ABA signaling. Responses to flooding or waterlogging involve ethylene signaling, which likely reduces plant resistance to chewing herbivores due to its negative cross-talk with JA. However, the outcome of interactions between biotic and abiotic stress signaling is often plant and/or insect species-dependent and cannot simply be predicted based on general knowledge on the involvement of signaling pathways in single stress responses. More experimental data on non-model plant and insect species are needed to reveal general patterns and better understand the molecular mechanisms allowing plants to optimize their responses in complex environments.

## Introduction

 Plants have to constantly cope with a suite of biotic and abiotic stress factors. Their performance thus depends on the ability to quickly perceive changes in the environment and to express an adaptive response. Much effort has been made to understand the molecular mechanisms underlying plant adaptive responses because of their potential to improve agricultural production under adverse conditions.

Plant molecular responses to single abiotic stresses, such as drought, soil flooding, high or low temperatures, as well as to biotic interactions, such as insect herbivory and pathogen attacks, have been gradually elucidated. These responses are modulated by a complicated network of signaling pathways induced by a variety of small molecules, including Ca^2+^ signaling (Seybold et al. 2014), reactive oxygen and nitrogen species (Wang et al. 2013; Baxter et al. 2014) and phytohormones (Peleg and Blumwald 2011; Pieterse et al. 2012; De Vleesschauwer et al. 2014; Kazan 2015). Hormones and hormonal cross-talk play an important role in the molecular mechanisms that optimize plant responses to stresses which commonly occur simultaneously in the environment, such as abiotic stresses and herbivory. Over the years several reviews have discussed cross-talk between defense-related hormonal pathways in plants challenged by different herbivores, different pathogens or combinations thereof (e.g. Pieterse et al. 2002; Erb et al. 2008; De Vleesschauwer et al. 2014). Independently, ecophysiologists acquired substantial knowledge on the role of hormonal signaling pathways in responses to abiotic stresses, such as drought, flooding and shading (e.g. Peleg and Blumwald 2011; Voesenek and Bailey-Serres 2015). Since long, several ecological studies revealed that (induced) resistance to herbivores can be affected by simultaneously occurring abiotic stresses, such as drought (English-Loeb et al. 1997; Huberty and Denno 2004; Khan et al. 2010; Gutbrodt et al. 2011; Tariq et al. 2013). However, only recently there has been an increased interest to identify the molecular mechanisms underlying these interactive effects (Lu et al. 2015; Davila Olivas et al. 2016; Foyer et al. 2016; Nguyen et al. 2016). For this reason, this a good moment for merging the knowledge on hormonal signaling in abiotic and biotic induced responses with the aim to come to a unified conceptual framework of how the signaling pathways induced by different stresses may interact. Thereby, we focus on the interactions between herbivore induced responses and water related stresses, specifically drought and flooding. Both drought and soil flooding or waterlogging are common phenomena in natural and agricultural ecosystems, and the frequency of their occurrence is expected to increase due to climate change (IPCC 2013). Here, we first review the most recent knowledge on how hormonal pathways regulate plant responses to single stresses. Then we discuss how interactions between these pathways may modulate defense responses in plants under combined stress conditions, considering that hormonal cross-talk may serve to optimize plant performance in complex environments. Finally, we will specify which testable hypotheses follow from our current knowledge that may help to better understand the role of signaling interactions in plants under multiple stresses.

## Regulation of induced plant responses to insect herbivores

**Fig. 1:**
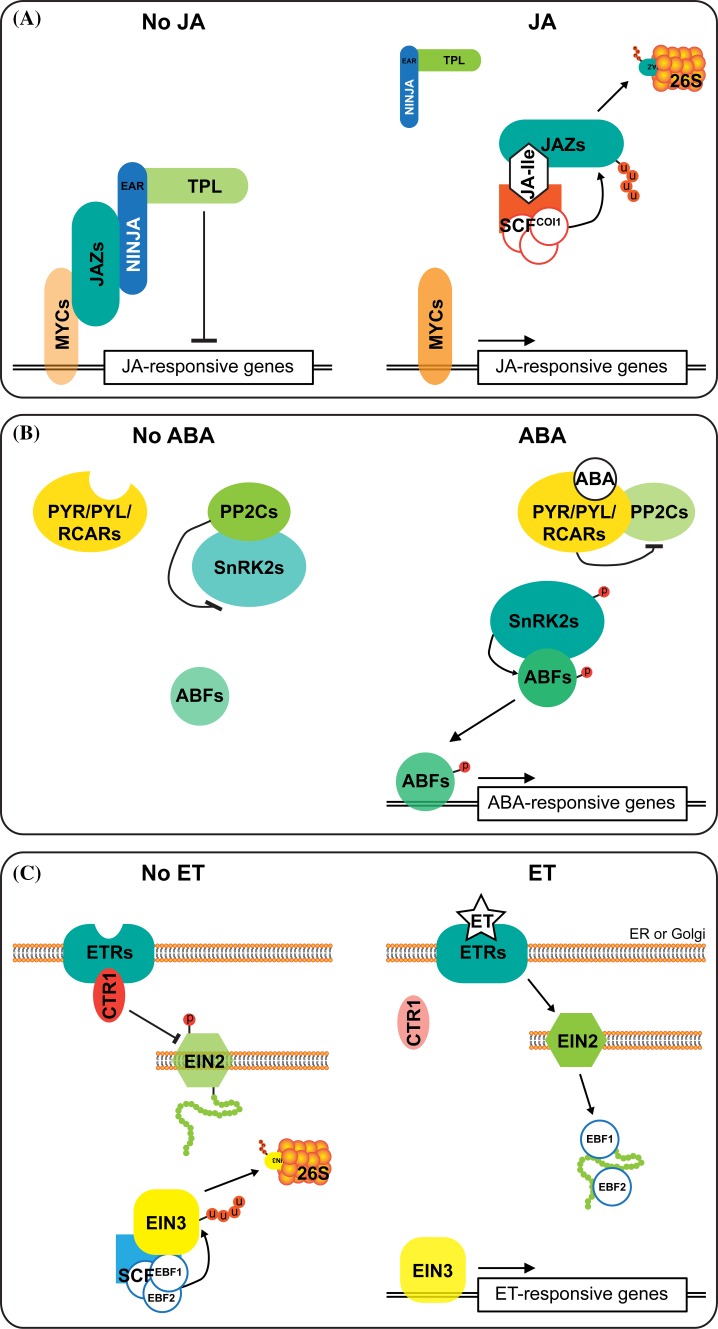
Schematic overview of hormonal signaling. **a** A model of jasmonic acid (JA) signaling, adapted from Pauwels et al. (2010). In the absence of JA, JAZs recruit the co-repressor TPL and TPRs via the EAR motif of the adaptor protein NINJA to suppress JA-responsive gene expression. This can also occur directly via the JAZ’s EAR motif (Shyu et al. 2012). In the presence of JA, JA-isoleucine conjugates are formed and facilitate the interaction between JAZs and SCF^COI1^, a multi-protein E3 ubiquitin ligase complex. This promotes JAZ ubiquitination and subsequent degradation by 26S proteasomes, resulting in the release of NINJA-TPL complex and activation of basic helix-loop-helix MYC transcription factors (TFs) to regulate JA-responsive genes. **b** A model for abscisic acid (ABA) signaling, adapted from Cutler et al. (2010). In the absence of ABA, PP2Cs are active to prevent SnRK2 activity. In the presence of ABA, PYR/PYL/RCARs bind to and inhibit PP2Cs, which allows phosphorylated SnRK2s to accumulate and subsequently phosphorylate ABFs to regulate ABA-responsive gene expression. **c** A model of ethylene (ET) signaling, adapted from Cho and Yoo (2014). In the absence of ET, the negative regulator CTR1 binds to membrane-bound ET receptors (ETRs) and inactivate the positive regulator EIN2. Moreover, the downstream primary TFs, EIN3 and EIL1, are constantly subjected to proteasomal degradation guided by EBF1 and EBF2. When ET has accumulated and binds to ET receptors, the ETR-CTR1 is inactivated. This leads to cleavage of C-terminal half of EIN2 and its translocation into nucleus to stabilize EIN3 by inactivating EBFs. EIN3 then regulates expression of downstream ET-responsive AP2/ERF TFs, such as ERF1 and ORA59

In natural habitats, plants have to defend themselves against herbivorous insects with different feeding strategies, including, but not limited to, leaf chewing beetles or caterpillars, piercing-sucking thrips or spider mites, and phloem-sucking aphids or whiteflies. Plant defense mechanisms may vary from morphological (e.g. trichomes, waxes) to chemical defenses [e.g. alkaloids, glucosinolates (GS), protease inhibitors (PIs)], which are often induced upon herbivory (Schaller 2008). When insects are feeding on plants, herbivore associated molecular patterns (HAMPs) and endogenous damage associated molecular patterns (DAMPS) are released (Acevedo et al. 2015). Upon perception of these cues, phytohormones, including jasmonic acid (JA), abscisic acid (ABA) and ethylene (ET), accumulate to activate signaling cascades that regulate downstream transcriptional responses (summarized in Fig. 1a–c). Among them, JA and particularly its most active isoleucine conjugate (JA-Ile), are generally accepted as the core inducers of many herbivore-induced defenses (Howe and Schaller 2008; Tytgat et al. 2013; Wasternack and Hause 2013). JA-insensitive or deficient mutants, therefore, exhibit very low levels of resistance to a wide range of herbivorous insects from different orders (Thaler et al. 2002; Bodenhausen and Reymond 2007; Schweizer et al. 2013).

Due to herbivore-specific HAMPs (Acevedo et al. 2015; Xu et al. 2015), other signaling hormones in addition to JA are induced upon feeding to tailor the defenses against the attacker. The signal signature that is induced for a part is due to differences in herbivore feeding strategies. Piercing-sucking insects, such as aphids, have a ‘stealthy feeding strategy’ (De Vos et al. 2005) that avoids massive cell damage. On the other hand, the salivary sheet lining their mandibles contains specific enzymes that interact with the cells along the stylet path (Foyer et al. 2016). Aphid feeding thus induces a significantly different set of signaling pathways and transcripts than chewing herbivores, that cause more cell damage and possess different elicitors in their saliva (De Vos et al. 2005; Bidart-Bouzat and Kliebenstein 2011). On the other hand, herbivore-induced signal signatures can also be species-specific within herbivore feeding guilds. For example, feeding by caterpillars of *Manduca sexta* induces the accumulation of JA and ET, whereas *Spodoptera exigua* caterpillars induce JA and salicylic acid (SA) in *Nicotiana attenuata* (Diezel et al. 2009). In contrast, *S. exigua* induces JA and ET accumulation in maize (*Zea mays*) and *Arabidopsis thaliana* (Schmelz et al. 2003; Rehrig et al. 2014), whereas *Pieris rapae* triggers JA and ABA levels in the latter species (Vos et al. 2013b). Simultaneous SA and JA accumulation also occurs upon herbivory by the Colorado potato beetle (*Leptinotarsa decemlineata*) and the mealy bug (*Phenacoccus solenopsis*) on tomato plants (*Solanum lycopersicum*) (Chung et al. 2013; Zhang et al. 2015a). Although not all hormones were measured in each study, this strongly suggests that plant hormonal responses to herbivores depend on the specific plant–insect interaction. Cross-talk between JA and other phytohormones has been proposed to fine-tune plant defense responses to specific attackers (Pieterse et al. 2012; Erb et al. 2012).

## ABA in defense regulation

**Fig. 2:**
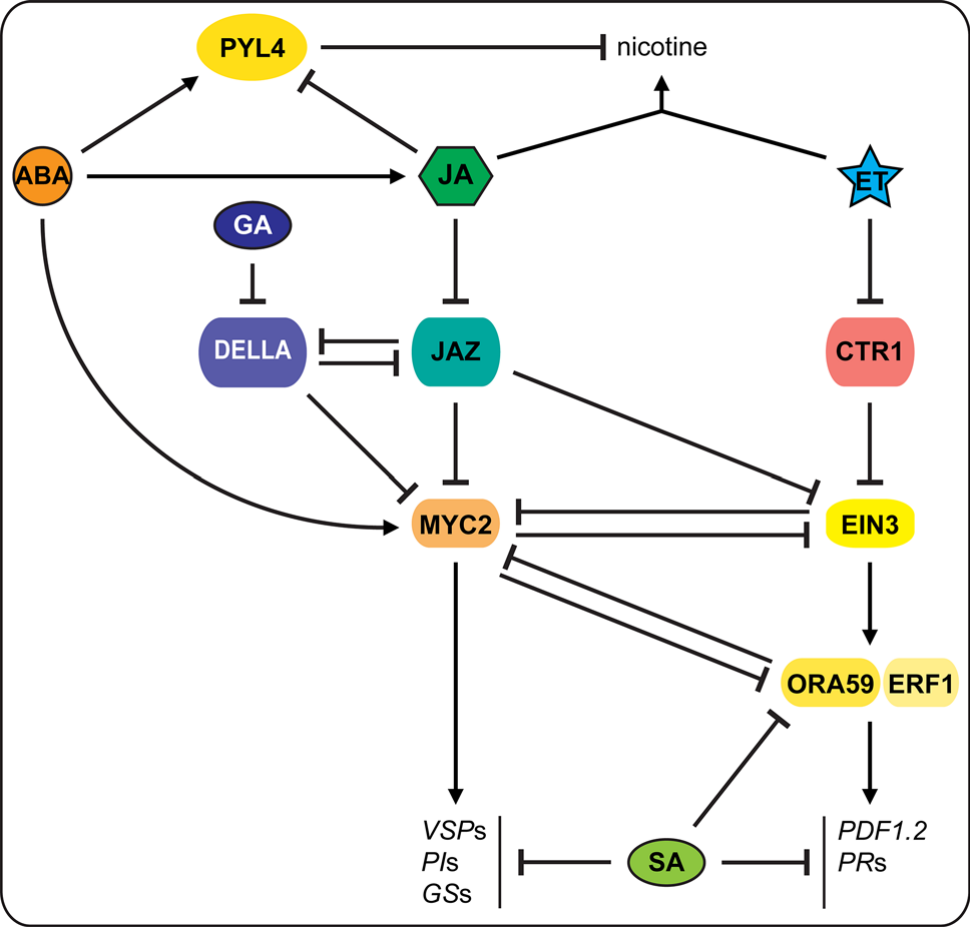
Schematic representation of interactions between hormonal cascades regulating induced defenses against biotic agents (see text and legend Fig. 1 for further details and abbreviations). Insect herbivores induce JA-dependent MYC2 regulation of defense-related genes, which is enahnced by ABA signaling. Necrotrophic pathogens induce JA/ET-dependent signaling to regulate ERF1 and ORA59 and downstream defense-related genes. The two branches of defense responses mutually antagonize one another. GA and SA signaling generally inhibit JA-dependent defense responses

ABA synthesis and signaling is required for plants, such as *Arabidopsis*, tomato and *N. attenuata*, to fully activate defenses and resistance against their herbivores; ABA deficiency increases plant susceptibility to herbivory (Thaler and Bostock 2004; Bodenhausen and Reymond 2007; Vos et al. 2013b; Dinh et al. 2013). Furthermore, ABA is involved in signaling process inducing JA-dependent defense responses in systemic tissues (Erb et al. 2009; Vos et al. 2013b). The synergistic interaction between JA and ABA can occur via the transcription factor (TF) MYC2 and its homologs MYC3 and MYC4 in *Arabidopsis* (Fig. 2). ABA induces COI-dependent expression of MYCs, which induce plant resistance to insects by regulating many wound/herbivore-responsive genes, e.g. *VSP*s, *LOX*s and glucosinolate biosynthetic genes (Lorenzo et al. 2004; Dombrecht et al. 2007; Schweizer et al. 2013). In tomato, the ABA/JA/wounding-responsive expression of *LAP* and the PI gene *PIN2* are directly regulated by MYC2 orthologs, JAMYC2 and JAMYC10 (Peña-Cortés et al. 1995; Boter et al. 2004). However, due to the strong mutual antagonism between ABA and ET, and the fact that some JA-responsive defenses are mediated by ET (discussed below), logically ABA also negatively affects some JA/ET-dependent defenses, such as nicotine biosynthesis in tobacco plants (*Nicotiana tabacum*) (Lackman et al. 2011).

A key question is where in the signaling cascades interactions between JA and ABA occur. The requirement of normal ABA biosynthesis for JA production (Adie et al. 2007), the COI-dependency of the ABA-induced MYC2 expression (Lorenzo et al. 2004) and the fact that methyljasmonate (MeJA) still induces *LAP* and *PIN2* in ABA-deficient mutants (Carrera and Prat 1998), suggest that the interaction occurs upstream of JA signaling. Indeed, JA and ABA mutually enhance their biosynthesis (Adie et al. 2007; Fan et al. 2009; Brossa et al. 2011). Mechanistic details on such interaction, however, are still lacking. Interestingly, it has been shown that interactions may also occur more downstream. A mechanism similar to the suppression of JA-induced TFs by JAZ–NINJA–TPL was identified for the ABA-dependent TF ABI5 (ABA insensitive5) in *Arabidopsis*. ABI5 binding proteins (AFPs) are NINJA homologs and contain the EAR motif to interact with the corepressors TPL or TPRs for ABI5 inactivation (Pauwels et al. 2010). Although the tested AFPs do not interact with JAZ1, this similarity nevertheless suggests that the JA–ABA interaction may exist at this JAZ–NINJA connection, downstream of JA biosynthesis, dependent on the binding specificity of different JAZs to NINJA or ABPs. This is supported by the recent finding that ZmJAZ14, a JAZ protein in maize, is involved in both JA and ABA signaling (Zhou et al. 2015).

## ET in defense regulation

Like JA, ET signaling upon feeding by insect herbivores is common among plants. However, ET has very variable effects on defense regulation, acting more as a modulator of herbivore-induced responses than a direct elicitor (von Dahl and Baldwin 2007). Very few plant defenses are directly regulated by ET. One known case is the induction of defensive 1-cysteine protease (Mir1-CP) against both chewing *Spodoptera frugiperda* and phloem-feeding *Rhopalosiphum maidis* in maize. JA also induces Mir1-CP expression upon *S. frugiperda* feeding, which is dependent on ET signaling, since MeJA treatment had no effect on Mir1-CP induction in maize plants with blocked ET signaling (Ankala et al. 2009; Louis et al. 2015). In many cases, ET has been shown to modulate JA-mediated insect defenses, similar to the well-documented ET–JA synergism in regulating defensive genes induced upon infestation by necrotrophic pathogens, such as *PDF1.2* and *PR1, 4 and 5*, via their co-regulation of the AP2/ERF TFs ERF1 and ORA59 (Lorenzo et al. 2003; Pré et al. 2008). For example, ET signaling contributes to the JA-mediated volatile emission upon *S. exigua* herbivory on maize or *Bemisia tabaci* infestation on *Arabidopsis* (Schmelz et al. 2003; Zhang et al. 2013). The wound-induced expression of tomato *PIN2* requires both intact JA and ET pathways, but compromising ET signaling does not affect the *M. sexta*-increased *PI* transcript levels in *N. attenuata* (O’Donnell et al. 1996; Onkokesung et al. 2010a). The complex involvement of ET in modulating herbivore/JA-induced defense responses also shows in nicotine biosynthesis. Defective ET signaling in *N. attenuata*, in one case, resulted in reduced basal nicotine contents but enhanced inducibility of nicotine biosynthesis after *M. sexta* herbivory (von Dahl et al. 2007), but in other experiments, it did not affect basal levels and attenuated JA-induced nicotine response (Shoji et al. 2000; Winz and Baldwin 2001; Onkokesung et al. 2010a). Nevertheless, both maize and *N. attenuata* with compromised ET signaling are more susceptible to *M. sexta* and *S. frugiperda*, respectively, demonstrating the role of ET in fortifying plant defenses (Harfouche et al. 2006; Onkokesung et al. 2010a). On the other hand, ET signaling, via ERF1/ORA59 and their upstream TFs EIN3/EIL1 (Fig. 1c), also inhibits the JA/ABA-co-induced MYC2 and subsequently MYC2-mediated defense-related genes in *Arabidopsis* (Lorenzo et al. 2004; Zhu et al. 2011; Song et al. 2014a). Consequently, disruptions of ET perception and signaling in *etr1*, *ein2*-*1* and *ein3/eil1* mutants all increase *Arabidopsis* resistance to the generalist insects *S. exigua* and *S. littoralis*, whereas ET application results in plant susceptibility. ET signaling, however, does not influence the responses and resistance of *Arabidopsis* to the specialists *Plutella xylostella* and *Pieris rapae* (Stotz et al. 2000; Mewis et al. 2005; Bodenhausen and Reymond 2007; Song et al. 2014a).

Recent findings also shed light on the mechanism of how these hormonal cascades interact (Fig. 2). Several JA signaling repressor JAZs bind to and inactivate EIN3/EIL1 and recruit HDA6 (histone deacetylase6) to repress EIN3/EIL1-dependent transcription (Zhu et al. 2011). Upon herbivore-induced ET and JA accumulation, ET signaling stabilizes EIN3/EIL1 while JAZ removal by JA signaling disassociates HDA6-EIN3/EIL1 and activates EIN3/EIL1 to transcribe downstream ERF1/ORA59. Interestingly, the ABA-inducible MYCs also physically interact with EIN3/EIL1, which mutually inhibits their function. Moreover, MYC2 indirectly promotes proteasomal degradation of EIN3 by enhancing EBF1 expression (Song et al. 2014a; Zhang et al. 2014). This illustrates how the balance between ABA and ET signaling fine-tunes JA-mediated defenses induced by insect herbivory.

## SA antagonizes herbivore-induced defenses

SA signaling mediates defense responses to hemi(biotrophic) pathogens (Derksen et al. 2013). This is achieved via its receptor and regulator NPR1 (nonexpressor of PR genes1) and the action of two NPR1 homologs, NPR3 and NPR4, which are also SA receptors and mediate NPR1 degradation in SA-concentration-dependent manners (Kuai et al. 2015). In some cases, SA-induced defense responses are effective against sedentary sucking insects, such as aphids (Klingler et al. 2009; Zhang et al. 2015b). SA accumulation in host plants can be induced by HAMPs and can also be exploited by insects to suppress JA-mediated defenses (Thaler et al. 2012; Caarls et al. 2015). Glucose oxidase in *S. exigua* oral secretion induces an SA burst in *N. attenuata*, which suppresses JA and ET accumulation (Diezel et al. 2009). Moreover, several insects carry viruses or microbes that trigger SA accumulation. *Tomato spotted wilt virus* transmitted by thrips feeding increases SA concentrations in *Arabidopsis*, resulting in increased performance and preference of thrips for infected plants (Abe et al. 2012). Flagellin from *Pseudomonas* sp. present on the mouth parts of *L. decemlineata* can induce SA accumulation in tomato leaves upon feeding, thereby suppressing JA-dependent defenses, such as PIs and polyphenol oxidases, and herbivore-induced resistance (Chung and Felton 2011; Chung et al. 2013).

The SA antagonism of JA-dependent defenses occurs downstream of JA biosynthesis and independently of the COI1-JAZs pathway. It inhibits defenses mediated by both ABA and ET signaling (Fig. 2). Disruption of SA accumulation or NPR1 function thus increases resistance to several chewing and sucking insects (Stotz et al. 2002; Mewis et al. 2005; Zarate et al. 2007). Cytosolic NPR1 activity is also a mediator of the SA–JA antagonism, which, however, is bypassed if herbivores also induce ET accumulation (Spoel et al. 2003; Leon-Reyes et al. 2009; Van der Does et al. 2013). Moreover, SA leads to degradation of the JA/ET-responsive ORA59 and suppresses JA/ET-responsive GCC-box-containing genes, including ORA59, by recruiting the SA-induced GRX480 (Glutaredoxin480) to their promoters. This inhibits the positive transcription regulators class II TGAs thereby repressing JA/ET-induced responses (Zander et al. 2012, 2014; Van der Does et al. 2013). Less is known about how SA inhibits JA/ABA-responsive defenses. Potential points of convergence in this interaction are WRKY TFs. WRKY62 and WRKY70 regulate the SA–JA antagonism in defense responses and ABA-responsive defense genes (Li et al. 2004; Mao et al. 2007), whereas WRKY18, WRKY40 and WRKY60 are ABA-responsive and blocked by SA (Xu et al. 2006; Chen et al. 2010).

## Growth hormones in defense regulation

**Fig. 3:**
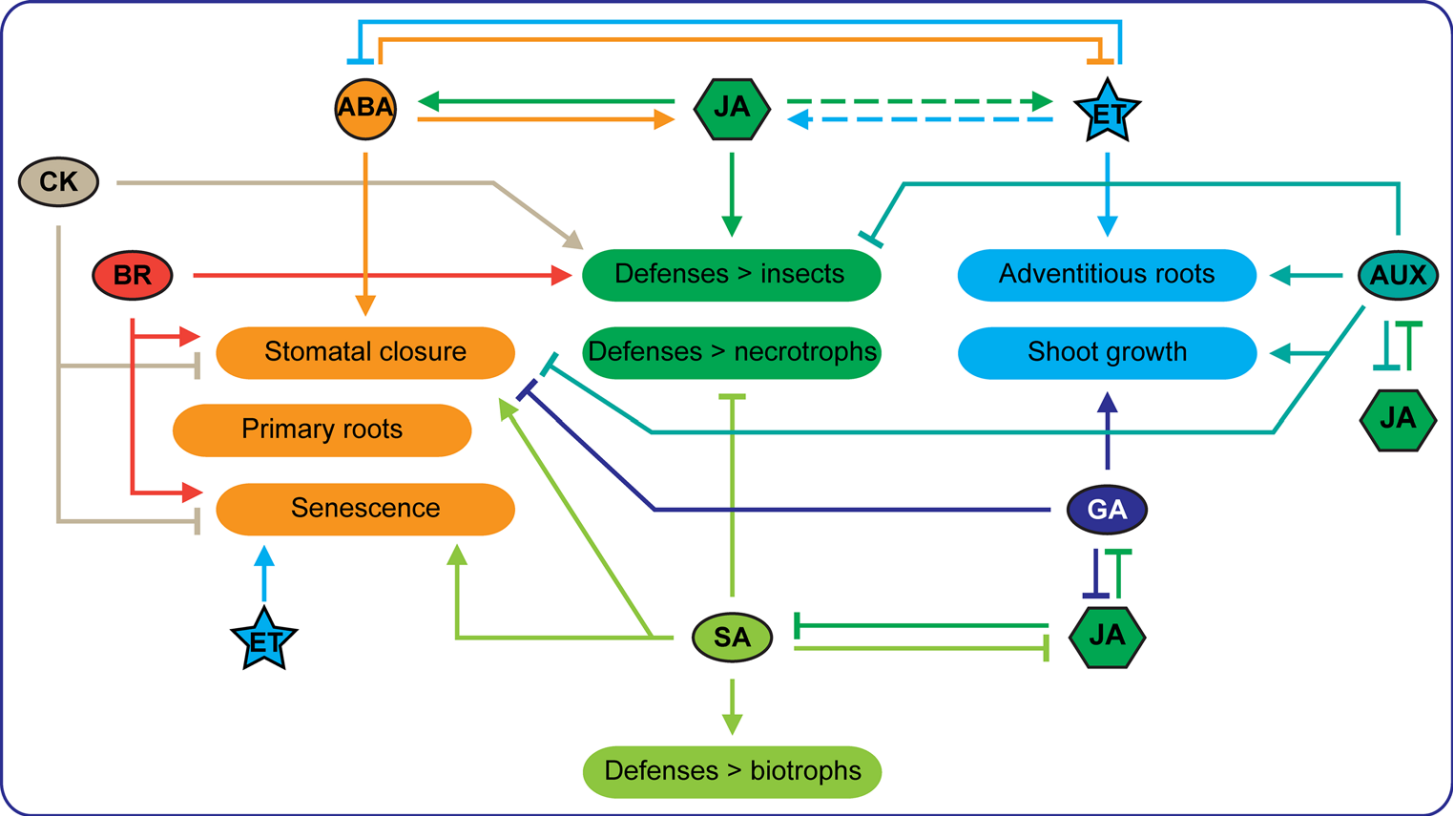
Hormonal interactions regulating plant responses to abiotic stresses and defenses against biotic agents.* Arrow heads* indicate a positive interaction, whereas a T end, indicates an inhibitory effect. Abscisic acid (ABA) has strong synergistic effects on JA-dependent defenses, while jasmonic acid (JA) promotes ABA-mediated stomatal closure and leaf senescence, but not primary root growth.* Dashed arrows* indicate the mixed effects of ethylene (ET) on JA-dependent defenses: ET induces defense responses to necrotrophic pathogens and some responses to insect herbivores but suppresses other insect induced defenses. ABA and ET strongly antagonize each other in many responses, but both induce leaf senescence. Interactions between JA and gibberellic acid (GA) or auxin (AUX) to mediate growth-defense balance are also indicated

Recently, phytohormones such as gibberellins (GAs), brassinosteroids (BRs), auxins (AUXs) and cytokinins (CKs) have also been shown to modulate JA-mediated responses to herbivores (Figs. 2, 3), besides their involvement in regulating defenses against pathogens (Naseem and Dandekar 2012; Denancé et al. 2013; De Bruyne et al. 2014). For example, GA signaling interacts with JA signaling via the negative regulators DELLAs. DELLAs and JAZs directly bind and deactivate each other (Fig. 2; Hou et al. 2013; Song et al. 2014b). In the presence of GA, DELLAs are degraded via the 26S proteasome, releasing JAZs to suppress MYC2 (Hou et al. 2010; Wild et al. 2012). On the other hand, DELLAs are necessary to attenuate *S. exigua*-induced JA accumulation in *Arabidopsis*, and consequently GA can promote JA biosynthesis (Cheng et al. 2009; Lan et al. 2014). Moreover, the DELLA protein RGA (repressor of GA1-3), binds to MYC2; its removal thus increases MYC2 activity (Hong et al. 2012). Another DELLA, RGL3 (RGA-like3), whose expression is enhanced by JA in a MYC2-dependent manner, can competitively bind to JAZs and further increase MYC2 activity (Wild et al. 2012). This JA-GA synergistic interaction plays a role in trichome initiation and sesquiterpene biosynthesis (Hong et al. 2012; Qi et al. 2014). Similarly, BRs, AUXs and CKs influence JA signaling both positively and negatively in regulating responses to herbivores (Dervinis et al. 2010; Yang et al. 2011; Meldau et al. 2011).

In conclusion, interactions between hormonal signaling cascades help plants to fine-tune their defenses against a specific attacker. Conversely, insects may have the ability to interfere with these hormonal interactions to suppress defense responses to their benefit.

## Hormonal regulation of plant responses to abiotic stresses

Due to its involvement in many developmental processes, such as shoot growth inhibition, stomatal movement, leaf senescence and primary root growth, ABA is considered as a master regulator of responses to abiotic stresses, such as drought, salt, heat and high light intensity (Fig. 3; Sharp et al. 2004; Daszkowska-Golec and Szarejko 2013; Liang et al. 2014). JA, SA and BRs also interact with ABA to promote stomatal closure, prevent water loss during osmotic stresses, and induce leaf senescence for resource remobilization (Hossain et al. 2011; Miura et al. 2012; Qi et al. 2015). Stomatal opening, on the other hand, is promoted by CKs and AUXs, while leaf senescence is inhibited by GAs, CKs and AUXs (Daszkowska-Golec and Szarejko 2013; Jibran et al. 2013). ET is also considered as a major inducer of leaf senescence (Kim et al. 2015), whereas ABA and ET show a clear antagonism in regulating stomatal movement (Tanaka et al. 2005) and shoot and root growth under drought (Fig. 3; Sharp and LeNoble 2002; Sharp et al. 2004; Yin et al. 2015). Similarly, ABA antagonizes ET in controlling flooding responses, such as shoot elongation, leaf hyponasty and adventitious root formation (Voesenek and Bailey-Serres 2015). The ET-mediated responses to flooding as well as shading, on the other hand, are synergistically regulated by GAs, BRs and AUXs (Cox et al. 2006; Gommers et al. 2013; van Veen et al. 2013; Pierik and Testerink 2014; Ayano et al. 2014). These insights demonstrate that plant responses to abiotic stresses and defense responses are controlled by the same interactive hormonal network.

## Hormonal interactions regulate growth-defense tradeoffs

The simultaneous roles of hormones in plant development and defense led to the view that they interact to prioritize resources towards growth or defense. This is a relevant concept when considering abiotic-biotic stress interaction, as abiotic stress usually severely impairs plant growth. The probability to survive under adverse conditions may increase if limited resources are efficiently allocated to tolerate abiotic stresses or to defend valuable tissues against herbivores (Van Dam and Baldwin 2001; Skirycz and Inzé 2010; Atkinson and Urwin 2012; Vos et al. 2013a). There is substantial evidence that this happens in case of pathogen attack (Denancé et al. 2013; Huot et al. 2014); and the regulation of the growth-defense tradeoff when plants are under combined abiotic stress and insect herbivory may also follow this strategy. The best illustrated hormonal interaction to regulate growth-defense tradeoffs is between JA and GA. Similar to their interaction in regulating defenses, JA also antagonizes GA-dependent growth responses via JAZs-DELLAs. In the absence of JA, *Arabidopsis* JAZ9 binds the DELLA protein RGA, thereby preventing it from inhibiting the growth promoting TF PIF3 (phytochrome-interacting factor3). Upon herbivory, JA induces JAZ degradation and delays GA-mediated DELLA degradation, allowing DELLAs to inhibit GA-dependent plant growth responses (Yang et al. 2012). Furthermore, JA in concert with ET repress cell cycle processes and expansion of leaf cells by suppressing the cell expansion enhancers, AUXs. Conversely, AUXs were proposed as repressors of JA synthesis and JA/ET-dependent nicotine response. AUXs and JA, however, synergistically constrain *N. attenuata* regrowth after *M. sexta* herbivory (Shi et al. 2006; Onkokesung et al. 2010b; Noir et al. 2013; Machado et al. 2013). ABA and JA signaling also synergistically suppress plant growth and yield under drought stress (Kim et al. 2009; Harb et al. 2010). On the other hand, ABA signaling antagonizes nicotine biosynthesis in *N. tabacum* roots via PYL4, an ABA receptor that controls root metabolic responses to drought and drought resistance; whereas JA suppresses *PYL4* expression in roots but enhances it in leaves (Fig. 2; Lackman et al. 2011; Pizzio et al. 2013; González-Guzmán et al. 2014). These examples show that the growth-defense balance is tightly regulated by a sophisticated network of hormonal cross-talk.

Furthermore, the growth-defense balance can also be controlled by master mediators that regulate multiple hormonal cascades. For example, the *Arabidopsis* CML42 (calmodulin-like protein42) suppresses both JA-dependent insect resistance and drought-responsive ABA accumulation; and the rice WRKY70 induces JA but represses GA biosynthesis and signaling (Vadassery et al. 2012; Li et al. 2015). However, the WRKY70-dependent prioritization of defenses over growth leads to resistance to the stem borer *Chilo suppressalis* but susceptibility to the brown planthopper *Nilaparvata lugens*, suggesting that defense prioritization is species-specific (Li et al. 2015).

## Hormonal regulation of defense responses under combined stresses

Despite our extensive knowledge on hormonal regulatory pathways and their interactions, predicting plant responses and phenotypes under combined biotic and abiotic stress remains difficult. Hormonal cascades may interact in non-additive manners and the results may enhance plant tolerance/resistance to one stress but not to another (Atkinson and Urwin 2012; Stam et al. 2014; Suzuki et al. 2014; Foyer et al. 2016). Also at the transcriptional level, stress combinations evoke responses that are unique or unpredictable from the responses to single stresses even if the points of convergence are known (Rasmussen et al. 2013; Atkinson et al. 2013). Abiotic stresses, such as drought, salt, heat or flooding, have been found to exert both positive and negative influences on resistance to pathogens and insect herbivores (DeLucia et al. 2012; Suzuki et al. 2014; Ramegowda and Senthil-Kumar 2015). For example, the strong JA-ABA synergism in many stress responses suggests that drought may promote plant resistance to herbivores. However, drought increases defense responses and render plants resistant to insect herbivores in some cases, but reduces defenses and resistance in others (English-Loeb et al. 1997; Huberty and Denno 2004; Khan et al. 2010; Gutbrodt et al. 2011; Tariq et al. 2013; Nguyen et al. 2016).

Recently, a few studies have tried to dissect hormonal interactions occurring under simultaneous abiotic stress and herbivory. In *Brassica oleracea* plants, drought and *Mamestra brassicae* herbivory interactively regulate the emission of volatile organic compounds (VOCs) as an indirect defense (Weldegergis et al. 2015). While drought alone induces SA accumulation and reduces the emissions of several VOCs, it also reduces herbivore-induced JA accumulation and consequently alters the herbivore-induced emissions of these VOCs. This resulted in *M. brassicae* moth preference to lay eggs on drought-stressed plants but no differences in larval performance compared to those on well-watered plants. Interestingly, ABA accumulation was observed upon herbivory but not in drought-stressed plants, possibly due to the intermittent drought stress regime with recovery periods, during which ABA catabolism may be induced (Wang 2002; Fleta-Soriano et al. 2015). In contrast, drought enhanced resistance of *Solanum dulcamara* plants to *S. exigua* larvae (Nguyen et al. 2016). Both dought and herbivory induced ABA and JA accumulation in *S. dulcamara*. Transcriptomic analyses showed drought further enhanced several herbivore-induced defense-related responses, such as terpenoid biosynthesis and PIs (Nguyen et al. 2016). Similarly, drought increased leaf ABA and JA concentrations, JA-dependent defense and *Medicago truncatula* plant resistance to the pea aphids *Acyrthosiphon pisum* (Gou et al. 2016). Therefore, the synergistic interaction between ABA and JA signaling is suggested to play an important role in regulating plant defense under drought. This is supported by the finding that ABA signaling is required for the full activation of VOC emission and JA-responsive direct defenses in *N. attenuata* (Dinh et al. 2013). Silencing of an ABA catabolism suppressor, NaHER1 (herbivore elicitor-regulated1), in *N. attenuata* resulted in reduced levels of these defense responses upon herbivory by *M. sexta* as well as plant resistance. Moreover, *NaHER1*-silenced plants are also drought-sensitive, suggesting that NaHER1 serves as a connection between responses to the two stresses.

On the other hand, there may be mechanisms underlying plant increased resistance to herbivores under drought that are independent of the ABA-JA signaling interaction. In maize, drought and root herbivory by *Diabrotica virgifera* synergistically enhance levels of ABA and ABA-dependent defense gene transcripts in the leaves and resistance to the leaf herbivore *Spodoptera littoralis* (Erb et al. 2011). However, leaf water loss, but not the induced ABA level itself, was strongly correlated to the resistance. Therefore, hydraulic changes induced by drought and root herbivory were suggested to play a role in inducing ABA/JA-independent signaling that increases resistance to above-ground herbivores.

Interestingly, there is much less knowledge on the effect of soil flooding on herbivore resistance, possibly because most model plants are crops and drought is more commonly recognized as a problem in production systems around the world than flooding or waterlogging. Only recently Lu et al. (2015) studied the hormonal interaction between flooding and root herbivory in rice. The study showed, however, that hormonal responses to root herbivory or artificial wounding was not altered by flooding. In *S. dulcamara*, soil flooding increased ABA, but not JA, levels in the leaves and suppressed many transcriptional responses involved in primary and secondary metabolism, including defense-related responses. These changes, however, did not affect the plant resistance to *S. exigua* larvae (Nguyen et al. 2016).

These insights, though not always as expected beforehand, are invaluable to understand how plants fine-tune their responses to specific combinations of stress conditions. Based on what we know about the interactions between hormones and a few experimental studies, we suggest that drought in general may enhance resistance because of the synergistic effect of ABA and JA signaling. Drought and herbivory both significantly reduce plant performance but when a drought period is followed by herbivory, the negative effect is more than additive (Davila Olivas et al. 2016). Thus it may be functional for a drought-stressed plant to reduce additional damage by increasing herbivore defenses. It should be stressed that the resulting effect on the herbivore may differ, depending on its level of host plant specialization or feeding strategy (Foyer et al. 2016). On the other hand, flooding or waterlogging elicits the production of ET. The interaction of ET with herbivore-induced responses is not as uniform as that found for ABA. Hence it can be expected that flooding has a neutral or negative effect on plant resistance. It is likely that plants surrounded by water (temporarily) do not receive as many herbivores as a plant on dry land. Therefore, it is conceivable that flooded plants may increase their performance more if they invest in overcoming the negative effects of hypoxia, for example by producing aerenchymous adventitious roots (Dawood et al. 2016).

## Conclusions

Simultaneously occurring stresses may compromise plant hormonal homeostasis. If this leads to a misregulation of stress responses, it may result in lower plant survival or yield reduction. Therefore, a better understanding of these hormonal interactions is essential to attain resilient and ‘multitasking’ crop plants that can perform well in adverse and variable environments. However, hormonal interactions under combined stresses cannot be simply inferred from experiments applying single stresses. Thus, more studies on plants responses to multiple and simultaneous stresses, especially abiotic stresses and insect herbivory, are needed to gain insights on how hormones truly interact under such—more natural—conditions. Furthermore, downstream changes induced by multiple stresses should be investigated by untargeted high-throughput approaches, such as transcriptomics, proteomics or metabolomics to obtain a broad and precise view of the regulatory and phenotypic consequences of hormonal interactions. Finally, plant performance or resistance should be assessed to validate the ecological effects of these molecular interactions. Given the common co-occurrence of abiotic and biotic stresses, the response to stress combination is likely to be under strong natural selection. Thus, we argue that the seemingly low level of conservation in the effect of abiotic stress on herbivore defenses, depending on plant and insect species, does not represent random output of the signaling network. Rather, it may be the consequence of divergent choices in prioritization and thus resource allocation that only appear upon combined stress application. Recognition of general patterns then requires availability of a larger set of data. Preferably, experiments should be carried out using plant species thriving in both wet and dry habitats as well as with a diverse natural herbivore community. This will allow us to ‘learn from nature’ whether plants can be selected to handle multiple stresses at the same time while maintaining a high performance.

## Abbreviations

ABF: ABA-responsive element binding factor
AP2/ERF: Apetala2/ethylene response factor
COI1: Coronatine insensitive1
CTR1: Constitutive triple response1
EAR: ERF-associated amphiphilic repression
EBF: EIN3-binding F-box protein
EIL1: EIN3-like protein1
EIN2: Ethylene insensitive2
EIN3: Ethylene insensitive3
JAZ: Jasmonate-ZIM domain corepressor
LAP: Leucine aminopeptidase
LOX: Lipoxygenase
NINJA: Novel interactor of JAZ
PDF1.2: Plant defensin
PP2C: Type 2C protein phosphatase
PR: Pathogenesis-related
PYR/PYL/RCAR: Pyrabactin resistance1/PYR-like/regulatory component of ABA receptor
SCF: Skp, Cullin, F-box containing
SnRK2: Sucrose non-fermenting1-related protein kinase2 protein
TPL: Groucho/Tup1-type co-repressor TOPLESS
TPR: TPL-related protein
VSP: Vegetative storage protein

## References

[CR1] Abe H, Tomitaka Y, Shimoda T, Seo S, Sakurai T, Kugimiya S, Tsuda S, Kobayashi M (2012). Antagonistic plant defense system regulated by phytohormones assists interactions among vector insect, thrips and a tospovirus. Plant Cell Physiol.

[CR2] Acevedo FE, Rivera-Vega LJ, Chung SH, Ray S, Felton GW (2015). Cues from chewing insects—the intersection of DAMPs, HAMPs, MAMPs and effectors. Curr Opin Plant Biol.

[CR3] Adie BAT, Pérez-Pérez J, Pérez-Pérez MM, Godoy M, Sánchez-Serrano J-J, Schmelz EA, Solano R (2007). ABA is an essential signal for plant resistance to pathogens affecting JA biosynthesis and the activation of defenses in *Arabidopsis*. Plant Cell.

[CR4] Ankala A, Luthe DS, Williams WP, Wilkinson JR (2009). Integration of ethylene and jasmonic acid signaling pathways in the expression of maize defense protein Mir1-CP. Mol Plant Microbe Interact.

[CR5] Atkinson NJ, Urwin PE (2012). The interaction of plant biotic and abiotic stresses: from genes to the field. J Exp Bot.

[CR6] Atkinson NJ, Lilley CJ, Urwin PE (2013). Identification of genes involved in the response of *Arabidopsis* to simultaneous biotic and abiotic stresses. Plant Physiol.

[CR7] Ayano M, Kani T, Kojima M, Sakakibara H, Kitaoka T, Kuroha T, Angeles-Shim RB, Kitano H, Nagai K, Ashikari M (2014). Gibberellin biosynthesis and signal transduction is essential for internode elongation in deepwater rice. Plant Cell Environ.

[CR8] Baxter A, Mittler R, Suzuki N (2014). ROS as key players in plant stress signalling. J Exp Bot.

[CR9] Bidart-Bouzat MG, Kliebenstein D (2011). An ecological genomic approach challenging the paradigm of differential plant responses to specialist versus generalist insect herbivores. Oecologia.

[CR10] Bodenhausen N, Reymond P (2007). Signaling pathways controlling induced resistance to insect herbivores in *Arabidopsis*. Mol Plant Microbe Interact.

[CR11] Boter M, Ruíz-Rivero O, Abdeen A, Prat S (2004). Conserved MYC transcription factors play a key role in jasmonate signaling both in tomato and *Arabidopsis*. Genes Dev.

[CR12] Brossa R, López-Carbonell M, Jubany-Marí T, Alegre L (2011). Interplay between abscisic acid and jasmonic acid and its role in water-oxidative stress in wild-type, ABA-deficient, JA-deficient, and ascorbate-deficient *Arabidopsis* plants. J Plant Growth Regul.

[CR13] Caarls L, Pieterse CMJ, Van Wees SCM (2015). How salicylic acid takes transcriptional control over jasmonic acid signaling. Front Plant Sci.

[CR14] Carrera E, Prat S (1998). Expression of the *Arabidopsis* abi1-1 mutant allele inhibits proteinase inhibitor wound-induction in tomato. Plant J.

[CR15] Chen H, Lai Z, Shi J, Xiao Y, Chen Z, Xu X (2010). Roles of *Arabidopsis* WRKY18, WRKY40 and WRKY60 transcription factors in plant responses to abscisic acid and abiotic stress. BMC Plant Biol.

[CR16] Cheng H, Song S, Xiao L, Soo HM, Cheng Z, Xie D, Peng J (2009). Gibberellin acts through jasmonate to control the expression of MYB21, MYB24, and MYB57 to promote stamen filament growth in *Arabidopsis*. PLoS Genet.

[CR17] Cho Y-H, Yoo S-D (2014). Novel connections and gaps in ethylene signaling from the ER membrane to the nucleus. Front Plant Sci.

[CR18] Chung SH, Felton GW (2011). Specificity of induced resistance in tomato against specialist lepidopteran and coleopteran species. J Chem Ecol.

[CR19] Chung SH, Rosa C, Scully ED, Peiffer M, Tooker JF, Hoover K, Luthe DS, Felton GW (2013). Herbivore exploits orally secreted bacteria to suppress plant defenses. Proc Natl Acad Sci USA.

[CR20] Cox MCH, Peeters AJM, Voesenek LACJ (2006). The stimulating effects of ethylene and auxin on petiole elongation and on hyponastic curvature are independent processes in submerged *Rumex palustris*. Plant Cell Environ.

[CR21] Cutler SR, Rodriguez PL, Finkelstein RR, Abrams SR (2010). Abscisic acid: emergence of a core signaling network. Annu Rev Plant Biol.

[CR22] Daszkowska-Golec A, Szarejko I (2013). Open or close the gate—stomata action under the control of phytohormones in drought stress conditions. Front Plant Sci.

[CR23] Davila Olivas NH, Coolen S, Huang P, Severing E, van Verk MC, Hickman R, Wittenberg AHJ, de Vos M, Prins M, van Loon JJA, Aarts MGM, van Wees SCM, Pieterse CMJ, Dicke M (2016). Effect of prior drought and pathogen stress on Arabidopsis transcriptome changes to caterpillar herbivory. New Phytol.

[CR24] Dawood T, Yang X, Visser EJW, te Beek TAH, Kensche PR, Cristescu SM, Lee S, Floková K, Nguyen D, Mariani C, Rieu I (2016). A co-opted hormonal cascade activates dormant adventitious root primordia upon flooding in *Solanum dulcamara*. Plant Physiol.

[CR25] De Bruyne L, Höfte M, De Vleesschauwer D (2014). Connecting growth and defense: the emerging roles of brassinosteroids and gibberellins in plant innate immunity. Mol Plant.

[CR26] De Vleesschauwer D, Xu J, Höfte M (2014). Making sense of hormone-mediated defense networking: from rice to *Arabidopsis*. Front Plant Sci.

[CR27] De Vos M, Van Oosten VR, Van Poecke RMP, Van Pelt JA, Pozo MJ, Mueller MJ, Buchala AJ, Metraux JP, Van Loon LC, Dicke M, Pieterse CMJ (2005). Signal signature and transcriptome changes of Arabidopsis during pathogen and insect attack. Mol Plant Microbe Interact.

[CR28] DeLucia EH, Nabity PD, Zavala JA, Berenbaum MR (2012). Climate change: resetting plant-insect interactions. Plant Physiol.

[CR29] Denancé N, Sánchez-Vallet A, Goffner D, Molina A (2013). Disease resistance or growth: the role of plant hormones in balancing immune responses and fitness costs. Front Plant Sci.

[CR30] Derksen H, Rampitsch C, Daayf F (2013). Signaling cross-talk in plant disease resistance. Plant Sci.

[CR31] Dervinis C, Frost CJ, Lawrence SD, Novak NG, Davis JM (2010). Cytokinin primes plant responses to wounding and reduces insect performance. J Plant Growth Regul.

[CR32] Diezel C, von Dahl CC, Gaquerel E, Baldwin IT (2009). Different lepidopteran elicitors account for cross-talk in herbivory-induced phytohormone signaling. Plant Physiol.

[CR33] Dinh ST, Baldwin IT, Galis I (2013). The HERBIVORE ELICITOR-REGULATED 1 gene enhances abscisic acid levels and defenses against herbivores in *Nicotiana attenuata* plants. Plant Physiol.

[CR34] Dombrecht B, Xue GP, Sprague SJ, Kirkegaard JA, Ross JJ, Reid JB, Fitt GP, Sewelam N, Schenk PM, Manners JM, Kazan K (2007). MYC2 differentially modulates diverse jasmonate-dependent functions in *Arabidopsis*. Plant Cell.

[CR35] English-Loeb G, Stout MJ, Duffey SS (1997). Drought stress in tomatoes: changes in plant chemistry and potential nonlinear consequences for insect herbivores. Oikos.

[CR36] Erb M, Ton J, Degenhardt J, Turlings TCJ (2008). Interactions between arthropod-induced aboveground and belowground defenses in plants. Plant Physiol.

[CR37] Erb M, Gordon-Weeks R, Flors V, Camañes G, Turlings TCJ, Ton J (2009). Belowground ABA boosts aboveground production of DIMBOA and primes induction of chlorogenic acid in maize. Plant Signal Behav.

[CR38] Erb M, Köllner TG, Degenhardt J, Zwahlen C, Hibbard BE, Turlings TCJ (2011). The role of abscisic acid and water stress in root herbivore-induced leaf resistance. New Phytol.

[CR39] Erb M, Meldau S, Howe GA (2012). Role of phytohormones in insect-specific plant reactions. Trends Plant Sci.

[CR40] Fan J, Hill L, Crooks C, Doerner P, Lamb C (2009). Abscisic acid has a key role in modulating diverse plant-pathogen interactions. Plant Physiol.

[CR41] Fleta-Soriano E, Pintó-Marijuan M, Munné-Bosch S (2015). Evidence of drought stress memory in the facultative CAM, *Aptenia cordifolia*: possible role of phytohormones. PLoS ONE.

[CR42] Foyer CH, Rasool B, Davey JW, Hancock RD (2016). Cross-tolerance to biotic and abiotic stresses in plants: a focus on resistance to aphid infestation. J Exp Bot.

[CR43] Gommers CMM, Visser EJW, St Onge KR, Voesenek LACJ, Pierik R (2013). Shade tolerance: when growing tall is not an option. Trends Plant Sci.

[CR44] González-Guzmán M, Rodríguez L, Lorenzo-Orts L, Pons C, Sarrión-Perdigones A, Fernández MA, Peirats-Llobet M, Forment J, Moreno-Alvero M, Cutler SR, Albert A, Granell A, Rodríguez PL (2014). Tomato PYR/PYL/RCAR abscisic acid receptors show high expression in root, differential sensitivity to the abscisic acid agonist quinabactin, and the capability to enhance plant drought resistance. J Exp Bot.

[CR45] Guo H, Sun Y, Peng X, Wang Q, Harris M, Ge F (2016). Up-regulation of abscisic acid signaling pathway facilitates aphid xylem absorption and osmoregulation under drought stress. J Exp Bot.

[CR46] Gutbrodt B, Mody K, Dorn S (2011). Drought changes plant chemistry and causes contrasting responses in lepidopteran herbivores. Oikos.

[CR47] Harb A, Krishnan A, Ambavaram MMR, Pereira A (2010). Molecular and physiological analysis of drought stress in *Arabidopsis* reveals early responses leading to acclimation in plant growth. Plant Physiol.

[CR48] Harfouche AL, Shivaji R, Stocker R, Williams PW, Luthe DS (2006). Ethylene signaling mediates a maize defense response to insect herbivory. Mol Plant Microbe Interact.

[CR49] Hong G-J, Xue X-Y, Mao Y-B, Wang L-J, Chen X-Y (2012). *Arabidopsis* MYC2 interacts with DELLA proteins in regulating sesquiterpene synthase gene expression. Plant Cell.

[CR50] Hossain MA, Munemasa S, Uraji M, Nakamura Y, Mori IC, Murata Y (2011). Involvement of endogenous abscisic acid in methyl jasmonate-induced stomatal closure in *Arabidopsis*. Plant Physiol.

[CR51] Hou X, Lee LYC, Xia K, Yan Y, Yu H (2010). DELLAs modulate jasmonate signaling via competitive binding to JAZs. Dev Cell.

[CR52] Hou X, Ding L, Yu H (2013). Crosstalk between GA and JA signaling mediates plant growth and defense. Plant Cell Rep.

[CR53] Howe GA, Schaller A, Schaller A (2008). Direct defenses in plants and their induction by wounding and insect herbivores. Induced plant resistance to herbivory.

[CR54] Huberty AF, Denno RF (2004). Plant water stress and its consequences for herbivorous insects: a new synthesis. Ecology.

[CR55] Huot B, Yao J, Montgomery BL, He SY (2014). Growth–defense tradeoffs in plants: a balancing act to optimize fitness. Mol Plant.

[CR56] IPCC (2013) Summary for policymakers. In: Stocker TF, D Qin, G-K Plattner, M Tignor, SK Allen, J Boschung, A Nauels, Y Xia, V Bex, PM Midgley (eds) Climate Change 2013: The Physical Science Basis. Contribution of Working Group I to the Fifth Assessment Report of the Intergovernmental Panel on Climate Change. Cambridge University Press, Cambridge

[CR57] Jibran R, Hunter A, Dijkwel P (2013). Hormonal regulation of leaf senescence through integration of developmental and stress signals. Plant Mol Biol.

[CR58] Kazan K (2015). Diverse roles of jasmonates and ethylene in abiotic stress tolerance. Trends Plant Sci.

[CR59] Khan MAM, Ulrichs C, Mewis I (2010). Influence of water stress on the glucosinolate profile of *Brassica oleracea var. italica* and the performance of *Brevicoryne brassicae* and *Myzus persicae*. Entomol Exp Appl.

[CR60] Kim EH, Kim YS, Park S-H, Koo YJ, Do Choi Y, Chung Y-Y, Lee I-J, Kim J-K (2009). Methyl jasmonate reduces grain yield by mediating stress signals to alter spikelet development in rice. Plant Physiol.

[CR61] Kim J, Chang C, Tucker ML (2015). To grow old: regulatory role of ethylene and jasmonic acid in senescence. Front Plant Sci.

[CR62] Klingler JP, Nair RM, Edwards OR, Singh KB (2009). A single gene, *AIN*, in *Medicago truncatula* mediates a hypersensitive response to both bluegreen aphid and pea aphid, but confers resistance only to bluegreen aphid. J Exp Bot.

[CR63] Kuai X, MacLeod BJ, Després C (2015). Integrating data on the *Arabidopsis* NPR1/NPR3/NPR4 salicylic acid receptors; a differentiating argument. Front Plant Sci.

[CR64] Lackman P, González-Guzmán M, Tilleman S, Carqueijeiro I, Pérez AC, Moses T, Seo M, Kanno Y, Häkkinen ST, Van Montagu MCE, Thevelein JM, Maaheimo H, Oksman-Caldentey K-M, Rodriguez PL, Rischer H, Goossens A (2011). Jasmonate signaling involves the abscisic acid receptor PYL4 to regulate metabolic reprogramming in *Arabidopsis* and tobacco. Proc Natl Acad Sci USA.

[CR65] Lan Z, Krosse S, Achard P, van Dam NM, Bede JC (2014). DELLA proteins modulate *Arabidopsis* defences induced in response to caterpillar herbivory. J Exp Bot.

[CR66] Leon-Reyes A, Spoel SH, De Lange ES, Abe H, Kobayashi M, Tsuda S, Millenaar FF, Welschen RAM, Ritsema T, Pieterse CMJ (2009). Ethylene modulates the role of NONEXPRESSOR OF PATHOGENESIS-RELATED GENES1 in cross talk between salicylate and jasmonate signaling. Plant Physiol.

[CR67] Li J, Brader G, Palva ET (2004). The WRKY70 transcription factor: a node of convergence for jasmonate-mediated and salicylate-mediated signals in plant defense. Plant Cell.

[CR68] Li R, Zhang J, Li J, Zhou G, Wang Q, Bian W, Erb M, Lou Y (2015). Prioritizing plant defence over growth through WRKY regulation facilitates infestation by non-target herbivores. Elife.

[CR69] Liang C, Wang Y, Zhu Y, Tang J, Hu B, Liu L, Ou S, Wu H, Sun X, Chu J, Chu C (2014). OsNAP connects abscisic acid and leaf senescence by fine-tuning abscisic acid biosynthesis and directly targeting senescence-associated genes in rice. Proc Natl Acad Sci USA.

[CR70] Lorenzo O, Piqueras R, Sánchez-Serrano JJ, Solano R (2003). ETHYLENE RESPONSE FACTOR1 integrates signals from ethylene and jasmonate pathways in plant defense. Plant Cell.

[CR71] Lorenzo O, Chico JM, Salchez-Serrano JJ, Solano R (2004). JASMONATE-INSENSITIVE1 Encodes a MYC transcription factor essential to discriminate between different jasmonate-regulated defense responses in *Arabidopsis*. Plant Cell.

[CR72] Louis J, Basu S, Varsani S, Castano-Duque L, Jiang V, Williams WP, Felton GW, Luthe DS (2015). Ethylene contributes to maize insect resistance 1-mediated maize defense against the phloem sap-sucking corn leaf aphid. Plant Physiol.

[CR73] Lu J, Robert CAM, Riemann M, Cosme M, Mène-Saffrané L, Massana J, Stout MJ, Lou Y, Gershenzon J, Erb M (2015). Induced jasmonate signaling leads to contrasting effects on root damage and herbivore performance. Plant Physiol.

[CR74] Machado RAR, Ferrieri AP, Robert CAM, Glauser G, Kallenbach M, Baldwin IT, Erb M (2013). Leaf-herbivore attack reduces carbon reserves and regrowth from the roots via jasmonate and auxin signaling. New Phytol.

[CR75] Mao P, Duan M, Wei C, Li Y (2007). WRKY62 transcription factor acts downstream of cytosolic NPR1 and negatively regulates jasmonate-responsive gene expression. Plant Cell Physiol.

[CR76] Meldau S, Baldwin IT, Wu J (2011). SGT1 regulates wounding- and herbivory-induced jasmonic acid accumulation and *Nicotiana attenuata*’s resistance to the specialist lepidopteran herbivore *Manduca sexta*. New Phytol.

[CR77] Mewis I, Appel HM, Hom A, Raina R, Schultz JC (2005). Major signaling pathways modulate *Arabidopsis* glucosinolate accumulation and response to both phloem-feeding and chewing insects. Plant Physiol.

[CR78] Miura K, Okamoto H, Okuma E, Shiba H, Kamada H, Hasegawa PM, Murata Y (2012). SIZ1 deficiency causes reduced stomatal aperture and enhanced drought tolerance via controlling salicylic acid-induced accumulation of reactive oxygen species in *Arabidopsis*. Plant J.

[CR79] Naseem M, Dandekar T (2012). The role of auxin-cytokinin antagonism in plant-pathogen interactions. PLoS Pathog.

[CR80] Nguyen D, D’Agostino N, Tytgat TOG, Sun P, Lortzing T, Visser EJW, Cristescu SM, Steppuhn A, Mariani C, van Dam NM, Rieu I (2016). Drought and flooding have distinct effects on herbivore-induced responses and resistance in Solanum dulcamara. Plant Cell Environ.

[CR81] Noir S, Bömer M, Takahashi N, Ishida T, Tsui T-L, Balbi V, Shanahan H, Sugimoto K, Devoto A (2013). Jasmonate controls leaf growth by repressing cell proliferation and the onset of endoreduplication while maintaining a potential stand-by mode. Plant Physiol.

[CR82] O’Donnell PJ, Calvert C, Atzorn R, Wasternack C, Leyser HMO, Bowles DJ (1996). Ethylene as a signal mediating the wound response of tomato plants. Science.

[CR83] Onkokesung N, Baldwin IT, Gális I (2010). The role of jasmonic acid and ethylene crosstalk in direct defense of *Nicotiana attenuata* plants against chewing herbivores. Plant Signal Behav.

[CR84] Onkokesung N, Gális I, von Dahl CC, Matsuoka K, Saluz H-P, Baldwin IT (2010). Jasmonic acid and ethylene modulate local responses to wounding and simulated herbivory in *Nicotiana attenuata* leaves. Plant Physiol.

[CR85] Pauwels L, Barbero GF, Geerinck J, Tilleman S, Grunewald W, Pérez AC, Chico JM, Vanden Bossche R, Sewell J, Gil E, García-Casado G, Witters E, Inzé D, Long JA, De Jaeger G, Solano R, Goossens A (2010). NINJA connects the co-repressor TOPLESS to jasmonate signalling. Nature.

[CR86] Peleg Z, Blumwald E (2011). Hormone balance and abiotic stress tolerance in crop plants. Curr Opin Plant Biol.

[CR87] Peña-Cortés H, Fisahn J, Willmitzer L (1995). Signals involved in wound-induced proteinase inhibitor II gene expression in tomato and potato plants. Proc Natl Acad Sci USA.

[CR88] Pierik R, Testerink C (2014). The art of being flexible: how to escape from shade, salt, and drought. Plant Physiol.

[CR89] Pieterse CMJ, van Wees SCM, Ton J, van Pelt JA, van Loon LC (2002). Signalling in rhizobacteria-induced systemic resistance in Arabidopsis thaliana. Plant Biol.

[CR90] Pieterse CMJ, Van der Does D, Zamioudis C, Leon-Reyes A, Van Wees SCM (2012). Hormonal modulation of plant immunity. Annu Rev Cell Dev Biol.

[CR92] Pizzio GA, Rodriguez L, Antoni R, Gonzalez-Guzman M, Yunta C, Merilo E, Kollist H, Albert A, Rodriguez PL (2013). The PYL4 A194T mutant uncovers a key role of PYR1-LIKE4/PROTEIN PHOSPHATASE 2CA interaction for abscisic acid signaling and plant drought resistance. Plant Physiol.

[CR93] Pré M, Atallah M, Champion A, De Vos M, Pieterse CMJ, Memelink J (2008). The AP2/ERF domain transcription factor ORA59 integrates jasmonic acid and ethylene signals in plant defense. Plant Physiol.

[CR94] Qi T, Huang H, Wu D, Yan J, Qi Y, Song S, Xie D (2014). *Arabidopsis* DELLA and JAZ proteins bind the WD-repeat/bHLH/MYB complex to modulate gibberellin and jasmonate signaling synergy. Plant Cell.

[CR95] Qi T, Wang J, Huang H, Liu B, Gao H, Liu Y, Song S, Xie D (2015). Regulation of jasmonate-induced leaf senescence by antagonism between bHLH subgroup IIIe and IIId factors in *Arabidopsis*. Plant Cell.

[CR96] Ramegowda V, Senthil-Kumar M (2015). The interactive effects of simultaneous biotic and abiotic stresses on plants: mechanistic understanding from drought and pathogen combination. J Plant Physiol.

[CR97] Rasmussen S, Barah P, Suarez-Rodriguez MC, Bressendorff S, Friis P, Costantino P, Bones AM, Nielsen HB, Mundy J (2013). Transcriptome responses to combinations of stresses in *Arabidopsis*. Plant Physiol.

[CR98] Rehrig EM, Appel HM, Jones AD, Schultz JC (2014). Roles for jasmonate- and ethylene-induced transcription factors in the ability of *Arabidopsis* to respond differentially to damage caused by two insect herbivores. Front Plant Sci.

[CR99] Schaller A (2008). Induced plant resistance to herbivory.

[CR100] Schmelz EA, Alborn HT, Banchio E, Tumlinson JH (2003). Quantitative relationships between induced jasmonic acid levels and volatile emission in *Zea mays* during Spodoptera exigua herbivory. Planta.

[CR101] Schweizer F, Fernández-Calvo P, Zander M, Diez-Diaz M, Fonseca S, Glauser G, Lewsey MG, Ecker JR, Solano R, Reymond P (2013). *Arabidopsis* basic helix-loop-helix transcription factors MYC2, MYC3, and MYC4 regulate glucosinolate biosynthesis, insect performance, and feeding behavior. Plant Cell.

[CR102] Seybold H, Trempel F, Ranf S, Scheel D, Romeis T, Lee J (2014). Ca^2+^ signalling in plant immune response: from pattern recognition receptors to Ca^2+^ decoding mechanisms. New Phytol.

[CR103] Sharp RE, LeNoble ME (2002). ABA, ethylene and the control of shoot and root growth under water stress. J Exp Bot.

[CR104] Sharp RE, Poroyko V, Hejlek LG, Spollen WG, Springer GK, Bohnert HJ, Nguyen HT (2004). Root growth maintenance during water deficits: physiology to functional genomics. J Exp Bot.

[CR105] Shi Q, Li C, Zhang F (2006). Nicotine synthesis in *Nicotiana tabacum* L. induced by mechanical wounding is regulated by auxin. J Exp Bot.

[CR106] Shoji T, Nakajima K, Hashimoto T (2000). Ethylene suppresses jasmonate-induced gene expression in nicotine biosynthesis. Plant Cell Physiol.

[CR107] Shyu C, Figueroa P, DePew CL, Cooke TF, Sheard LB, Moreno JE, Katsir L, Zheng N, Browse J, Howe GA (2012). JAZ8 lacks a canonical degron and has an EAR motif that mediates transcriptional repression of jasmonate responses in *Arabidopsis*. Plant Cell.

[CR108] Skirycz A, Inzé D (2010). More from less: plant growth under limited water. Curr Opin Biotechnol.

[CR109] Song S, Huang H, Gao H, Wang J, Wu D, Liu X, Yang S, Zhai Q, Li C, Qi T, Xie D (2014). Interaction between MYC2 and ETHYLENE INSENSITIVE3 modulates antagonism between jasmonate and ethylene signaling in *Arabidopsis*. Plant Cell.

[CR110] Song S, Qi T, Wasternack C, Xie D (2014). Jasmonate signaling and crosstalk with gibberellin and ethylene. Curr Opin Plant Biol.

[CR111] Spoel SH, Koornneef A, Claessens SMC, Korzelius JP, Van Pelt JA, Mueller MJ, Buchala AJ, Métraux J-P, Brown R, Kazan K, Van Loon LC, Dong X, Pieterse CMJ (2003). NPR1 modulates cross-talk between salicylate- and jasmonate-dependent defense pathways through a novel function in the cytosol. Plant Cell.

[CR112] Stam JM, Kroes A, Li Y, Gols R, van Loon JJA, Poelman EH, Dicke M (2014). Plant interactions with multiple insect herbivores: from community to genes. Annu Rev Plant Biol.

[CR113] Stotz HU, Pittendrigh BR, Kroymann J, Weniger K, Fritsche J, Bauke A, Mitchell-Olds T (2000). Induced plant defense responses against chewing insects. Ethylene signaling reduces resistance of *Arabidopsis* against Egyptian cotton worm but not diamondback moth. Plant Physiol.

[CR114] Stotz H, Koch T, Biedermann A, Weniger K, Boland W, Mitchell-Olds T (2002). Evidence for regulation of resistance in *Arabidopsis* to Egyptian cotton worm by salicylic and jasmonic acid signaling pathways. Planta.

[CR115] Suzuki N, Rivero RM, Shulaev V, Blumwald E, Mittler R (2014). Abiotic and biotic stress combinations. New Phytol.

[CR116] Tanaka Y, Sano T, Tamaoki M, Nakajima N, Kondo N, Hasezawa S (2005). Ethylene inhibits abscisic acid-induced stomatal closure in *Arabidopsis*. Plant Physiol.

[CR117] Tariq M, Wright DJ, Bruce TJA, Staley JT (2013). Drought and root herbivory interact to alter the response of above-ground parasitoids to aphid infested plants and associated plant volatile signals. PLoS ONE.

[CR118] Thaler JS, Bostock RM (2004). Interactions between abscisic-acid-mediated responses and plant resistance to pathogens and insects. Ecology.

[CR119] Thaler JS, Farag M, Paré PW, Dicke M (2002). Jasmonate-deficient plants have reduced direct and indirect defences against herbivores. Ecol Lett.

[CR120] Thaler JS, Humphrey PT, Whiteman NK (2012). Evolution of jasmonate and salicylate signal crosstalk. Trends Plant Sci.

[CR121] Tytgat TOG, Verhoeven KJF, Jansen JJ, Raaijmakers CE, Bakx-Schotman T, McIntyre LM, van der Putten WH, Biere A, van Dam NM (2013). Plants know where it hurts: root and shoot jasmonic acid induction elicit differential responses in *Brassica oleracea*. PLoS ONE.

[CR122] Vadassery J, Reichelt M, Hause B, Gershenzon J, Boland W, Mithöfer A (2012). CML42-mediated calcium signaling coordinates responses to *Spodoptera* herbivory and abiotic stresses in *Arabidopsis*. Plant Physiol.

[CR123] van Dam NM, Baldwin IT (2001). Competition mediates costs of jasmonate-induced defences, nitrogen acquisition and transgenerational plasticity in *Nicotiana attenuata*. Funct Ecol.

[CR124] van der Does D, Leon-Reyes A, Koornneef A, Van Verk MC, Rodenburg N, Pauwels L, Goossens A, Körbes AP, Memelink J, Ritsema T, Van Wees SCM, Pieterse CMJ (2013) Salicylic acid suppresses jasmonic acid signaling downstream of SCF^COI1^-JAZ by targeting GCC promoter motifs via transcription factor ORA59. Plant Cell tpc.112.108548. doi: 10.1105/tpc.112.108548 10.1105/tpc.112.108548 PMC3608790 23435661

[CR125] van Veen H, Mustroph A, Barding GA, Vergeer-van Eijk M, Welschen-Evertman RAM, Pedersen O, Visser EJW, Larive CK, Pierik R, Bailey-Serres J, Voesenek LACJ, Sasidharan R (2013). Two Rumex species from contrasting hydrological niches regulate flooding tolerance through distinct mechanisms. Plant Cell.

[CR126] Voesenek LACJ, Bailey-Serres J (2015). Flood adaptive traits and processes: an overview. New Phytol.

[CR127] von Dahl CC, Baldwin IT (2007). Deciphering the role of ethylene in plant–herbivore interactions. J Plant Growth Regul.

[CR128] von Dahl CC, Winz RA, Halitschke R, Kühnemann F, Gase K, Baldwin IT (2007). Tuning the herbivore-induced ethylene burst: the role of transcript accumulation and ethylene perception in *Nicotiana attenuata*. Plant J.

[CR129] Vos IA, Pieterse CMJ, van Wees SCM (2013). Costs and benefits of hormone-regulated plant defences. Plant Pathol.

[CR130] Vos IA, Verhage A, Schuurink RC, Watt LG, Pieterse CMJ, Van Wees SCM (2013). Onset of herbivore-induced resistance in systemic tissue primed for jasmonate-dependent defenses is activated by abscisic acid. Front Plant Sci.

[CR131] Wang Z (2002). Abscisic acid catabolism in maize kernels in response to water deficit at early endosperm development. Ann Bot.

[CR132] Wang Y, Loake GJ, Chu C (2013). Cross-talk of nitric oxide and reactive oxygen species in plant programed cell death. Front Plant Sci.

[CR133] Wasternack C, Hause B (2013). Jasmonates: biosynthesis, perception, signal transduction and action in plant stress response, growth and development. An update to the 2007 review in Annals of Botany. Ann Bot.

[CR134] Weldegergis BT, Zhu F, Poelman EH, Dicke M (2015). Drought stress affects plant metabolites and herbivore preference but not host location by its parasitoids. Oecologia.

[CR135] Wild M, Davière J-M, Cheminant S, Regnault T, Baumberger N, Heintz D, Baltz R, Genschik P, Achard P (2012). The *Arabidopsis* DELLA RGA-LIKE3 is a direct target of MYC2 and modulates jasmonate signaling responses. Plant Cell.

[CR136] Winz RA, Baldwin IT (2001). Molecular interactions between the specialist herbivore *Manduca sexta* (Lepidoptera, Sphingidae) and its natural host *Nicotiana attenuata*. IV. Insect-Induced ethylene reduces jasmonate-induced nicotine accumulation by regulating putrescine N-methyltransfer. Plant Physiol.

[CR137] Xu X, Chen C, Fan B, Chen Z (2006). Physical and functional interactions between pathogen-induced *Arabidopsis* WRKY18, WRKY40, and WRKY60 transcription factors. Plant Cell.

[CR138] Xu S, Zhou W, Pottinger S, Baldwin IT (2015). Herbivore associated elicitor-induced defences are highly specific among closely related *Nicotiana* species. BMC Plant Biol.

[CR139] Yang D-H, Hettenhausen C, Baldwin IT, Wu J (2011). BAK1 regulates the accumulation of jasmonic acid and the levels of trypsin proteinase inhibitors in *Nicotiana attenuata’*s responses to herbivory. J Exp Bot.

[CR140] Yang D-L, Yao J, Mei C-S, Tong X-H, Zeng L-J, Li Q, Xiao L-T, Sun T, Li J, Deng X-W, Lee CM, Thomashow MF, Yang Y, He Z, He SY (2012). Plant hormone jasmonate prioritizes defense over growth by interfering with gibberellin signaling cascade. Proc Natl Acad Sci USA.

[CR141] Yin C-C, Ma B, Collinge DP, Pogson BJ, He S-J, Xiong Q, Duan K-X, Chen H, Yang C, Lu X, Wang Y-Q, Zhang W-K, Chu C-C, Sun X-H, Fang S, Chu J-F, Lu T-G, Chen S-Y, Zhang J-S (2015). Ethylene responses in rice roots and coleoptiles are differentially regulated by a carotenoid isomerase-mediated abscisic acid pathway. Plant Cell.

[CR142] Zander M, Chen S, Imkampe J, Thurow C, Gatz C (2012). Repression of the *Arabidopsis thaliana* jasmonic acid/ethylene-induced defense pathway by TGA-interacting glutaredoxins depends on their C-terminal ALWL motif. Mol Plant.

[CR143] Zander M, Thurow C, Gatz C (2014). TGA transcription factors activate the salicylic acid-suppressible branch of the ethylene-induced defense program by regulating ORA59 expression. Plant Physiol.

[CR144] Zarate SI, Kempema LA, Walling LL (2007). Silverleaf whitefly induces salicylic acid defenses and suppresses effectual jasmonic acid defenses. Plant Physiol.

[CR145] Zhang P-J, Broekgaarden C, Zheng S-J, Snoeren TAL, van Loon JJA, Gols R, Dicke M (2013). Jasmonate and ethylene signaling mediate whitefly-induced interference with indirect plant defense in *Arabidopsis thaliana*. New Phytol.

[CR146] Zhang X, Zhu Z, An F, Hao D, Li P, Song J, Yi C, Guo H (2014). Jasmonate-activated MYC2 represses ETHYLENE INSENSITIVE3 activity to antagonize ethylene-promoted apical hook formation in *Arabidopsis*. Plant Cell.

[CR147] Zhang P-J, Huang F, Zhang J-M, Wei J-N, Lu Y-B (2015). The mealybug *Phenacoccus solenopsis* suppresses plant defense responses by manipulating JA–SA crosstalk. Sci Rep.

[CR148] Zhang X, Xue M, Zhao H (2015). Species-specific effects on salicylic acid content and subsequent *Myzus persicae* (Sulzer) performance by three phloem-sucking insects infesting *Nicotiana tabacum* L. Arthropod Plant Interact.

[CR149] Zhou X, Yan S, Sun C, Li S, Li J, Xu M, Liu X, Zhang S, Zhao Q, Li Y, Fan Y, Chen R, Wang L (2015). A maize jasmonate Zim-domain protein, ZmJAZ14, associates with the JA, ABA, and GA signaling pathways in transgenic *Arabidopsis*. PLoS ONE.

[CR150] Zhu Z, An F, Feng Y, Li P, Xue L, Jiang Z, Kim J-M, To TK, Li W, Zhang X, Yu Q, Dong Z, Chen W-Q, Seki M, Zhou J-M, Guo H (2011). Derepression of ethylene-stabilized transcription factors (EIN3/EIL1) mediates jasmonate and ethylene signaling synergy in *Arabidopsis*. Proc Natl Acad Sci USA.

